# Bullying Trends or Definitional Drift? A Methodological Critique of Molcho et al.’s Study

**DOI:** 10.3389/ijph.2025.1608711

**Published:** 2025-06-02

**Authors:** Romain Brisson

**Affiliations:** Sociodicy Institute, Corbenay, France

**Keywords:** bullying, temporal trends, definitional inconsistency, methodological considerations, HBSC study

To the editor,

Molcho et al. recently investigated temporal trends in bullying using data from the *Health Behaviour in School-aged Children* (HBSC) survey [1]. The authors analyzed the evolution of in-school bullying (1994–2022) and cyberbullying (2018–2022) prevalence. Results were indicative of a decrease in in-school bullying perpetration and victimization and of an increase in cyberbullying perpetration and victimization. While I commend the authors’ efforts to provide insights into temporal trends in bullying, I have two main concerns about the validity of their findings.

First, while the authors acknowledged that cyberbullying is an ill-defined construct [[1], p. 2], they omitted to mention that the same applies to in-school or so-called traditional bullying [2–5]. Notably, the authors did not clarify how (cyber)bullying has been defined in the HBSC study and did not reproduce the preamble outlining bullying in each HBSC survey. It appears, however, that this preamble has changed over time. As an illustration, in 1998, this preamble stated:

We say a student is being bullied when another student, or a group of students, say or do nasty and unpleasant things to him or her. It is also bullying when a student is teased repeatedly in a way he or she doesn’t like. But it is not bullying when two students of about the same strength quarrel or fight [[6], p. 2095].

I note that the reference to repetitiveness is made in the second sentence in relation to teasing. In 2018, the preamble stated:

We say a person is being bullied when another person or a group of people, repeatedly say or do unwanted nasty and unpleasant things to him or her. It also is bullying when a person is teased in a way he or she does not like or when he or she is left out of things on purpose. The person that bullies has more power than the person being bullied and wants to cause harm to him or her. It is not bullying when two people of about the same strength or power argue or fight [[7], p. 2].

Here, the reference to repetitiveness is made in the first sentence and no longer relates to teasing. This modification involved a different conception of bullying that may *partly* account for *some* observed changes in the prevalence of in-school bullying victimization and perpetration. In effect, having nasty and unpleasant things said or done to someone *repeatedly* over the past couple of months may occur less frequently than *a single instance* of such an event. In addition, in contrast to its 2018 version, the preamble used in 1998 mentioned neither social bullying (i.e., being “left out of things on purpose”) nor bully’s intent to harm. Coupled with the changes, reported by the authors [[1], p. 3], applied to the response rating scales, these definitional revisions question the diachronic comparability of the data examined in this study.

Second, whereas the authors aimed to examine temporal trends in in-school bullying perpetration and victimization, they excluded from their analyses the participants reporting either having been bullied or having bullied schoolmates once or twice in the past couple of months. The authors justified their use of this categorization criterion by (a) referring to a study by Sollberg and Olweus [8] and (b) claiming that doing so permitted them to “capture a regular pattern of perpetration [and victimization]” [[1], p. 3]. I do not think these are sound arguments. Indeed, Sollberg and Olweus used a definitional preamble in which repetitiveness was not a necessary dimension of bullying but only a *potential* characteristic of the phenomenon [[8], p. 246]. Because the preamble used in the most recent version of the HBSC survey attributes to repetitiveness an essential role, both frameworks should not be considered as conveying the same definition of bullying. For instance, reporting having been insulted once can refer to a one-off event within Sollberg and Olweus’ framework; by contrast, the “same” report refers to one sequence of *repeated* insults within the HBSC study’s framework. Unfortunately, the HBSC survey assesses neither the number of repetitions nor the time scope of the sequence(s) in question. In sum, invoking Sollberg and Olweus’ study is unwarranted, since Molcho et al. relied on a different conceptual and methodological design; claiming that the categorization criterion employed allowed the authors to “capture regular patterns” is misleading, since they did not assess the number of repetitions and/or the duration of the entire bullying sequence(s).

Considered altogether, these two points question the validity of the study findings. Temporal trend analysis requires the use of consistent assessment methods over time. Crucially, Molcho et al.’s study does not meet this basic requirement. Moreover, the exclusion of the participants reporting having been bullied or having bullied schoolmates once or twice in the past couple of months rests on problematic theoretical and methodological grounds. While including these participants might only marginally impact the overall trends, it would significantly affect prevalence estimates. I therefore urge readers to interpret the study findings with caution and advise against adopting the same categorization criterion in future research using data from the HBSC survey.
